# Epidemiological Characteristics of Primary Liver Cancer in Mainland China From 2003 to 2020: A Representative Multicenter Study

**DOI:** 10.3389/fonc.2022.906778

**Published:** 2022-06-21

**Authors:** Jiansheng Lin, Hongwei Zhang, Hongping Yu, Xinyu Bi, Weilu Zhang, Jianhua Yin, Pei Zhao, Xiumei Liang, Chunfeng Qu, Minjie Wang, Ming Hu, Kun Liu, Yuting Wang, Zihan Zhou, Junqi Wang, Xiaojie Tan, Wenbin Liu, Zhongjun Shao, Jianqiang Cai, Weizhong Tang, Guangwen Cao

**Affiliations:** ^1^ Department of Epidemiology, Second Military Medical University, Shanghai, China; ^2^ Department of Epidemiology, School of Medicine, Jinan University, Guangzhou, China; ^3^ Department of Research, Guangxi Medical University Cancer Hospital, Nanning, China; ^4^ Department of Hepatobiliary Surgery, National Cancer Center/National Clinical Research Center for Cancer/Cancer Hospital, Chinese Academy of Medical Sciences and Peking Union Medical College, Beijing, China; ^5^ Department of Epidemiology, School of Public Health, Fourth Military Medical University, Xi’an, China; ^6^ Office for Disease Process Management, Guangxi Medical University Cancer Hospital, Nanning, China; ^7^ State Key Lab of Molecular Oncology, National Cancer Center/National Clinical Research Center for Cancer/Cancer Hospital, Chinese Academy of Medical Sciences and Peking Union Medical College, Beijing, China; ^8^ Department of Clinical Laboratory, National Cancer Center/National Clinical Research Center for Cancer/Cancer Hospital, Chinese Academy of Medical Sciences and Peking Union Medical College, Beijing, China; ^9^ Guangxi Office for Cancer Prevention and Control, Guangxi Medical University Cancer Hospital, Nanning, China; ^10^ Department of Gastrointestinal Surgery and Guangxi Clinical Research Center for Colorectal Cancer, Guangxi Medical University Cancer Hospital, Nanning, China

**Keywords:** primary liver cancer, hepatocellular carcinoma, hepatitis B virus, hepatitis C virus, prognosis

## Abstract

**Background:**

The contribution of hepatitis B virus (HBV) and hepatitis C virus (HCV) to primary liver cancer (PLC) and their association with cancer aggressiveness remains uncertain in China, a country with half of global PLC. We aimed to characterize this using data from four representative medical centers.

**Methods:**

In total, 15,801 PLC patients were enrolled from the centers distributed in Easter5n, Southern, Northern, and Western China from 2003 to 2020. Of those, 7585 with curative surgery were involved in survival analysis. A nomogram was constructed using preoperative parameters to predict postoperative survival.

**Results:**

Hepatocellular carcinoma (HCC), intrahepatic cholangiocarcinoma, and combined hepatocellular cholangiocarcinoma accounted for 93.0%, 4.3%, and 1.6% in PLC, respectively. The seropositivities of HBV and HCV were 84.4% and 3.2% in HCC, respectively. The seropositivity of anti-HCV antibody was significantly higher in HBV-negative than in HBV-positive HCC patients (13.2% vs. 1.1%). Compared to HCV-positive HCC (HCV-HCC), HBV-positive HCC (HBV-HCC) was associated with 12-year earlier onset, higher proportions of males, high α-fetoprotein, large tumor size, advanced Barcelona Clinic Liver Cancer (BCLC) stage, and vascular tumor thrombus. The proportions of HCC and HBV seropositivity increased, whereas that of anti-HCV decreased, from 2003 to 2020. Postoperative five-year survival rate was 73.5%, 64.1%, 34.9%, and 19.7% in HCC at BCLC stage 0, A, B, and C, respectively. The multivariate Cox regression analysis showed that HBV seropositivity, incomplete tumor capsule, vascular tumor thrombus, tumor diameter (≥3 cm), advanced BCLC stage (B+C), α-fetoprotein (≥20ng/ml), and direct bilirubin (>8µmol/L) contributed independently to shorter overall survival (OS); whereas post-operative radiofrequency ablation and second resection independently improved OS in HCC. HCV-HCC had a more favorable prognosis than did HBV-HCC (Log-rank test, *P*<0.001). A nomogram composed of age, gender, and the preoperative independent risk factors was accurate in predicting postoperative survival in HCC (C-index: 0.735; 95% confidence interval: 0.727–0.743).

**Conclusion:**

HBV contributes to 84.4% of HCC in China, and actively promotes hepatocarcinogenesis and HCC progression. A favorable postoperative survival obtained in patients at the early BCLC stage highlights the importance of screening for early HCC in high-risk populations. Our preoperative prognosis prediction model is important in clinical decision-making.

## Introduction

Cancer may surpass cardiovascular disease as the leading cause of immature death in 57 countries including China (1). Primary liver cancer (PLC) is the sixth most commonly diagnosed cancer and the third leading cause of cancer death worldwide in 2020, with 905,677 new cases and 830,180 deaths in 2020 (2). PLC remains the second cause of cancer death and the first leading cause of immature cancer death in mainland China (2–4). The major histotypes of PLC are hepatocellular carcinoma (HCC), intrahepatic cholangiocarcinoma (ICC), and combined hepatocellular cholangiocarcinoma (CHC). The major causes of HCC are chronic infection with hepatitis B virus (HBV) and/or hepatitis C virus (HCV), alcohol consumption, aflatoxin B1 exposure, non-alcoholic fatty liver disease (NAFLD), and diabetes (5, 6). The risk factors of ICC include primary sclerosing cholangitis, hepatolithiasis, infection with *Opisthorchis viverrini* and *Clonorchis sinensis*, and chronic infection with HBV or HCV (7). However, the proportions of etiological agents and histotypes of PLC differ greatly among different studies. It was reported that 56% of global PLC were attributable to HBV and 20% to HCV (5); however, the corresponding proportions were estimated to be 33% and 21% in another global study (8). HCV infection is the leading cause of HCC in most European and American countries, while chronic HBV infection is the leading etiologic factor of HCC in Asian and African countries where HCC is endemic (5). HCC comprised 75%-85% while ICC 10%-15% of global PLC (2); however, HCC and ICC comprised 94.6% and 3.7% of PLC in eastern China (9). These data reflect apparent heterogeneities in the major etiological agents and the major histotypes of PLC worldwide.

In China, the contribution of major etiological agents to PLC and the proportion of major histotypes were only from very limited resources (9, 10). Currently, there are no representative data describing the major etiological agents and the proportion of major histotypes of PLC in China, a country with half of global PLC. A large, highly representative study population is indispensable to address these issues. The effect of HBV or HCV infection on the prognosis of HCC remains obscure. It has been shown that HBV-related HCC (HBV-HCC) has a better prognosis than HCV-related HCC (HCV-HCC) (11–13). However, this result is not repeated in other populations (14). This issue should be addressed by the propensity score (PS) matching method. In this study, we firstly evaluated the seropositivities of HBV and HCV in large-scale PLC patients from four representative medical centers in mainland China, and then investigated the difference in clinical characteristics of HBV-HCC and HCV-HCC. Finally, we developed a nomogram composed of preoperative clinical parameters to predict postoperative prognosis in HCC.

## Materials and Methods

### Participants Enrollment

In total, 15,816 consecutive patients with PLC were enrolled from four medical centers located in Northern (Beijing), Eastern (Shanghai), Western (Wuwei, Gansu), and Southern (Nanning, Guangxi) parts of mainland China from January 1, 2003, to June 30, 2020. Of those, 13,978 cases were pathologically diagnosed. Post-operative cohort studies were established based on personal willingness for the analysis of risk factors related to survival. The follow-up was carried out after curative surgery according to our existing protocol (15). During the follow-up, the information on the survival situation, the exact date of death, and treatment(s) received after surgery were collected. If patients had imaging evidence of tumor recurrence, second resection or radiofrequency ablation (RFA) was suggested (16). Postoperative transcatheter arterial chemoembolization (TACE) was recommended for patients with microvascular invasion (MVI) as previously described (17).

### Data Collection

The data of demographic and clinical characteristics were extracted from medical records, including birth date, age of onset, gender, nationality, place of birth, pathological findings (including pathological types, capsule integrity of tumor, nodule number, and vascular invasion), and laboratory examinations (serum AFP, parameters of HBV and HCV, routine blood assay, and liver function test). Barcelona Clinic Liver Cancer (BCLC) stage was identified as previously described (18). Han Chinese accounted for 91.2% of the study participants. The study protocol conformed to the ethical guidelines of the 2000 Declaration of Helsinki and was approved by the ethics committee of each involved medical center.

### Statistical Analysis

Data were independently checked by two researchers carefully. Fifteen duplicated cases were removed from the analysis. Those seropositive for HBsAg and/or HBV DNA were defined as positive HBV infection. The seropositive cutoff values for HBsAg and HBV DNA were >0.05 IU/mL and >500 copies/mL, respectively. The cutoff values of AFP, total bilirubin, direct bilirubin, and albumin were in accordance with the criteria adopted in clinic examination. For categorical variables, χ^2^ test or Fisher’s exact test was conducted for comparison between groups. Continuous variables with skewed distribution were compared by Mann–Whitney *U*-test. The trends of change in proportion of variables, such as HBV, HCC, and BCLC, were tested by using the Cochran-Armitage method. A Cox proportional hazard model was conducted to calculate the azard ratio (HR) and 95% confidence interval (CI) for each variable. Significant variables in the univariate Cox analysis and professionally meaningful variables were introduced into the multivariate Cox model, and the backward stepwise Wald method was applied to determine the factors that independently contributed to postoperative survival. The 1:2 propensity score (PS) matching was performed for survival comparison between HCV-HCC and HBV-HCC patients. For the comparison of the prognosis between HBV-HCC and HCV-HCC patients, Kaplan–Meier method was conducted to estimate overall survival (OS), and log-rank test was applied to compare the difference of OS between the two groups. For the prediction of HCC prognoses, the cohort was randomly grouped into training and validation sub-cohorts. A Cox regression model composed of statistically significant preoperative variables was established in the training, following the Akaike information criterion (19). A nomogram with these preoperative variables was formulated by the *rms* package in R (20). The fitness of the nomogram was evaluated by the concordance index (C-index) and calibration plots with 1000 bootstraps. The prediction power of the nomogram was verified in the validation cohort. Time-dependent receiver operating characteristics (ROC) curve was also applied to evaluate the accuracy of the nomogram in the training cohort and validation cohort. According to the medium of risk score calculated from the established nomogram, the subjects were divided into low-risk and high-risk groups. The difference in OS between the two groups was compared in the training cohort and validation cohort, respectively. All statistical analyses were two-sided and performed using SPSS V21.0 for Windows (http://www-01.ibm.com/software/uk/analytics/spss/,RRID:SCR_002865) and R software (version 4.0.2, https://www.r-project.org/). α*=*0.05 was considered astatistically significant level.

## Results

### Characteristics of Patients With PLC

In total, 15,816 PLC cases admitted to the four medical centers from January 1, 2003, to June 30, 2020 were enrolled in this study. The chart flow of study patients is shown in **Supplementary Figure 1**. Enrolled patients were from almost all provinces of mainland China. Interestingly, patients enrolled in Beijing were frequently from Heilongjiang province, while patients in Shanghai were frequently from nearby provinces (**Supplementary Figure 2**). The medium age of 15,801 patients at the diagnosis of PLC was 54 (inter-quartile range [IQR], 46–62 years). A male-to-female ratio was 5.29:1. Among all PLC patients, 80.1% were seropositive for HBV and 3.4% were seropositive for anti-HCV antibody. The seropositivity for HBV was more frequent in the east (87.6%) and south (87.7%) than in the north (57.2%) and west (61.6%), which was quite in contrast to the seropositivity for anti-HCV antibody (**Table 1**). Male PLC patients were 2 years younger than female patients at diagnosis (54 [46-62] vs. 56 [48-63] years, *P*<0.001). Compared to female PLC patients, males had higher proportions of advanced BCLC stage, liver cirrhosis, abnormal total bilirubin, and direct bilirubin. The seropositivity for HBV was more frequent in male than in female PLC patients (81.7% vs. 71.0%, *P*<0.001), which is in contrast to the seropositivity for anti-HCV antibody (3.2% vs. 4.4%) (**Supplementary Table 1**). Of 15,801 PLC patients, 13,978 had pathological data. HCC, ICC, and CHC accounted for 93.0%, 4.3%, and 1.6%, respectively. HCC, ICC, and CHC accounted for 94.3%, 3.3%, and 1.5% in the males and 85.7%, 10.5%, and 2.0% in the female PLC patients, respectively. Patients with HCC were 3 years younger than patients with ICC at diagnosis (53 [46-61] vs. 57 [49-64], *P*<0.001). The rate of seropositivity for HBV was 84.4%, 38.6%, and 77.1% while that for anti-HCV antibody was 3.2%, 1.8%, and 1.5% in patients with HCC, patients with ICC, and those with CHC, respectively.

**Table 1:** Demographic and clinical characteristics of PLC patients from the four medical centers.

Variable	Total(n = 15801)	Shanghai(n = 8515)	Beijing(n = 2561)	Nanning(n = 2813)	Wuwei(n = 1912)	P
**Age (yr)**						
Medium (IQR)	54 (46–62)	54 (46–61)	56 (48–63)	51 (43–60)	58 (49-66)	<0.001
<40	1,562 (9.9)	780 (9.2)	206 (8.0)	443 (15.7)	133 (7.0)	<0.001
40–59	9,098 (57.6)	5,113 (60.0)	1,424 (55.6)	1,664 (59.2)	897 (46.9)	
≥60	5,141 (32.5)	2,622 (30.8)	931 (36.4)	706 (25.1)	882 (46.1)	
**Gender**						
Female	2,519 (15.9)	1,191 (14.0)	468 (18.3)	367 (13.0)	493 (25.8)	<0.001
Male	13,282 (84.1)	7,324 (86.0)	2,093 (81.7)	2,446 (87.0)	1,419 (74.2)	
**HBV**						
Negative	2,999 (19.9)	1,034 (12.4)	1,095 (42.8)	345 (12.3)	525 (38.4)	<0.001
Positive	12,064 (80.1)	7,293 (87.6)	1,461 (57.2)	2,467 (87.7)	843 (61.6)	
**HCV**						
Negative	11,027 (96.6)	7,860 (98.3)	1,941 (91.1)	739 (98.1)	487 (91.5)	<0.001
Positive	386 (3.4)	137 (1.7)	190 (8.9)	14 (1.9)	45 (8.5)	
**Cirrhosis**						
No	7,357 (53.8)	4,684 (55.2)	500 (59.2)	1,088 (39.5)	1,085 (68.4)	<0.001
Yes	6,318 (46.2)	3,803 (44.8)	345 (40.8)	1,668 (60.5)	502 (31.6)	
**AFP (ng/ml)**						
<20	4,940 (39.3)	3,187 (37.9)	820 (57.3)	933 (34.2)	–	<0.001
≥20	7,618 (60.7)	5,211 (62.1)	611 (42.7)	1,796 (65.2)	–	
**Albumin (g/L)**						
≥40	6,661 (69.6)	5,577 (68.2)	164 (48.7)	920 (34.5)	–	<0.001
<40	4,518 (40.4)	2,595 (31.8)	173 (51.3)	1,750 (65.5)	–	
**Total bilirubin (µmol/L)**						
≤23	10,077 (88.9)	7,530 (90.5)	315 (92.1)	2,232 (83.6)	–	<0.001
>23	1,257 (11.1)	792 (9.5)	27 (7.9)	438 (16.4)	–	
**Direct bilirubin (µmol/L)**						
≤8	8,976 (81.5)	7,034 (84.5)	–	1,942 (72.2)	–	<0.001
>8	2,035 (18.5)	1,288 (15.5)	–	747 (27.8)	–	
**Ascites**						
No	11,205 (96.7)	8,370 (98.4)	474 (95.0)	2,361 (91.6)	–	<0.001
Yes	381 (3.3)	140 (1.6)	25 (5.0)	216 (8.4)	–	
**BCLC stage**						
0	472 (4.0)	378 (4.5)	69 (8.9)	25 (1.0)	–	<0.001
A	4,906 (41.3)	3,028 (35.9)	633 (81.7)	1,245 (48.5)	–	
B	4,330 (36.7)	3,699 (43.8)	57 (7.3)	574 (22.4)	–	
C	2,076 (17.6)	1,336 (15.8)	16 (2.1)	724 (28.2)	–	
**Pathological type**						
HCC	13,003 (93.0)	8,056 (94.6)	2,110 (82.4)	2,759 (98.3)	78 (83.0)	<0.001
ICC	607 (4.3)	314 (3.7)	282 (11.0)	0	11 (11.7)	
CHC	222 (1.6)	145 (1.7)	59(2.3)	16 (0.6)	2 (2.1)	
Others	146 (1.0)	0	110 (4.3)	33 (1.2)	3 (3.2)	

Data are shown in n (%).

### Trends in the Seropositivities of HBV and HCV, the Proportions of HCC and Early-Stage Tumors as Well as Postoperative Survival in PLC Patients From 2003 to 2020

**Figure 1:**
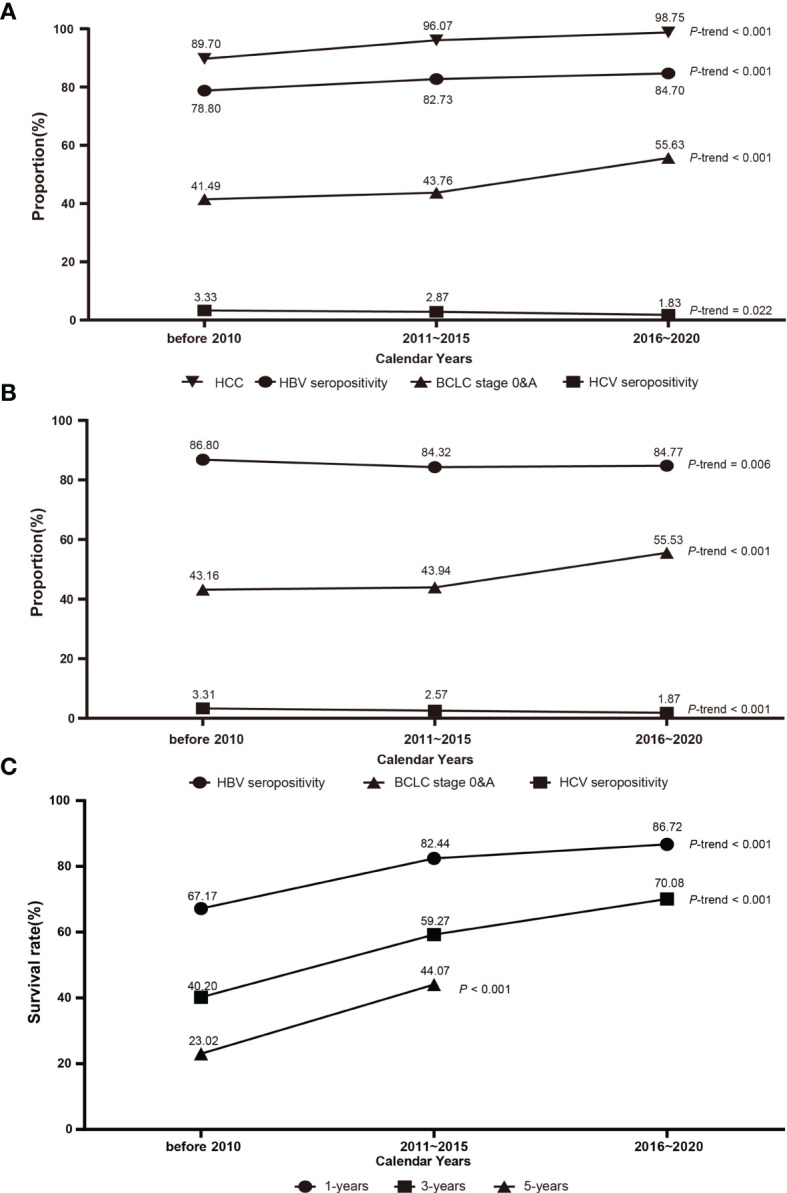
The dynamic trend of clinical parameters of PLC patients from 2003 to 2020. **(A)** The proportions of HCC, HBV seropositivity, HCV seropositivity, and early BCLC stage in PLC. **(B)** The proportions of HBV seropositivity, HCV seropositivity, and early BCLC stage in HCC. **(C)** The dynamic trends of 1-year, 3-year, and 5-year survival rates. Linear trends were calculated by using Cochran-Armitage test. BCLC, Barcelona Clinic Liver Cancer; HBV, hepatitis B virus; HCV, hepatitis C virus; HCC, hepatocellular carcinoma.

From 2003 to 2020, the seropositivity of HBV as well as the proportions of HCC and early BCLC stage (0&A) showed increasing trends in PLC patients (**Figure 1A**). However, the seropositivity of HBV in HCC patients declined from 86.8% before 2010 to 84.32% in 2011-2015 (**Figure 1B**). The seropositivity for anti-HCV antibody decreased significantly either in all PLC or in HCC (**Figures 1A, B**). Interestingly, the postoperative survival including 1-year survival and 3-year survival rates showed increasing trends in HCC patients from 2003 to 2020; the 5-year survival rate had a similar tendency from 2003 to 2015 (**Figure 1C**, *P*_trend_ < 0.001).

### Clinical Characteristics Between PLC Patients With HBV Infection and Those Without HBV Infection

Compared to HCC patients without HBV infection, those with HBV infection were 8 years younger and showed higher proportions of AFP (≥20 ng/ml), the presence of liver cirrhosis, abnormal albumin (<40g/L), advanced BCLC stage, multiple tumor nodules, and vascular tumor thrombus as well as a higher male-to-female ratio. The seropositivity for anti-HCV antibody in HBV-free HCC patients was significantly higher than that in those with HBV infection (13.2% vs. 1.1%, *P*<0.001) (**Table 2**). ICC patients with HBV infection were 5 years younger and had a higher male-to-female ratio, higher proportions of AFP (≥20ng/ml), liver cirrhosis, incomplete tumor capsule, vascular tumor thrombus, and advanced BCLC stage compared to those without HBV infection (**Supplementary Table 2**). We then compared the clinical characteristics of HCC patients solely caused by HBV infection and those by HCV infection. Compared to HCV-HCC patients, HBV-HCC patients were 12 years younger and had a higher proportion in males, advanced BCLC stage, vascular tumor thrombus, multiple tumor nodules, larger tumor size (≥3 cm in diameter), and higher AFP (≥20ng/mL) (**Table 3**).

**Table 2:** Comparison of demographic and clinical characteristics in HCC patients with information on HBV infection.

Variable	Total (n = 12,824)	Patients without HBV infection (n = 2,001)	Patients with HBV infection (n = 10,823)	*P*-value
**Age (year)**				
Medium (IQR)	53 (46-61)	60 (53-68)	52 (45-60)	
<40	1,356 (10.6)	103 (5.1)	1,253 (11.6)	<0.001
40–59	7,639 (59.6)	822 (41.1)	6,817 (63.0)	
≥60	3,829 (29.8)	1,076 (53.8)	2,753 (25.4)	
**Gender**				
Female	1,718 (13.4)	302 (15.1)	1,416 (13.1)	0.015
Male	11,106 (86.6)	1,699 (84.9)	9,407 (86.9)	
**HCV**				
Negative	9,710 (96.9)	1,480 (86.8)	8,230 (98.9)	<0.001
Positive	315 (3.1)	225 (13.2)	90 (1.1)	
**Cirrhosis**				
No	5,762 (50.7)	1,052 (67.9)	4,710 (48.0)	<0.001
Yes	5,610 (49.3)	498 (32.1)	5,112 (52.0)	
**Ascites**				
No	10,488 (96.6)	1,336 (95.5)	9,152 (96.8)	0.016
Yes	370 (3.4)	63 (4.5)	307 (3.2)	
**BCLC stage**				
0	451 (3.7)	47 (3.2)	404 (4.2)	<0.001
A	4,670 (39.6)	694 (47.7)	3,976 (41.4)	
B	4,018 (38.0)	518 (35.6)	3,500 (36.4)	
C	1,926 (18.7)	197 (13.5)	1,729 (18.0)	
**Tumor thrombus**				
No	6,812(65.0)	875 (68.1)	5,937 (64.5)	0.011
Yes	3,670(35.0)	409 (31.9)	3,261 (35.5)	
**Tumor nodule**				
Single	8,084 (80.1)	987 (84.2)	7,097 (79.6)	<0.001
Multiple	2,003 (19.9)	185 (15.8)	1,818 (20.4)	
**Tumor diameter (cm)**				
<3	1,644 (12.7)	175 (10.6)	1,298 (12.7)	0.007
≥3	9,950 (87.3)	1,469 (89.4)	8,652 (87.3)	
**Tumor capsule**				
Yes	7,129 (70.8)	830 (70.6)	6,299 (70.8)	0.867
No	2,942 (29.2)	346 (29.4)	2,596 (29.2)	
**AFP (ng/ml)**				
<20	4,417 (37.6)	800 (49.9)	3,617 (35.6)	<0.001
≥20	7,334 (62.4)	803 (50.1)	6,531 (64.4)	
**Total bilirubin (µmol/L)**				
≤23	9,483 (88.9)	1,152 (88.5)	8,331 (88.9)	0.635
>23	1,188 (11.1)	150 (11.5)	1,038 (11.1)	
**Direct bilirubin (µmol/L)**				
≤8	8,444 (81.5)	965 (81.4)	7,479 (81.5)	0.976
>8	1,921 (18.5)	220 (18.6)	1,701 (18.5)	
**Albumin (g/L)**				
≥40	6,555 (59.6)	912 (62.8)	5,643 (59.1)	0.007
<40	4,451 (40.4)	541 (37.2)	3,910 (40.9)	

Data are shown in n (%).

**Table 3:** Comparison of demographic and clinical characteristics between HBV- and HCV-related HCC patients.

Variable	Total (n = 8,455)	Patients with HBV positive only (n = 8,230)	Patients with HCV positive only (n = 225)	*P-*value
**Age (year)**				
Medium (IQR)	52 (45-60)	52 (45-60)	64 (57-71)	
<40	864 (10.2)	861 (10.5)	3 (1.3)	<0.001
40–59	5,274 (62.4)	5,207 (63.3)	67 (29.8)	
≥60	2,317 (27.4)	2,162 (26.2)	155 (68.9)	
**Gender**				
Female	1,128 (13.3)	1,083 (13.2)	45 (20.0)	0.003
Male	7,327 (86.7)	7,147 (86.8)	180 (80.0)	
**Cirrhosis**				
No	3,906 (51.2)	3,838 (51.2)	68 (51.5)	0.945
Yes	3,720 (48.8)	3,656 (48.8)	64 (48.5)	
**Ascites**				
No	7,249 (97.9)	7,147 (98.0)	102 (94.4)	0.011
Yes	155 (2.1)	149 (2.0)	6 (5.6)	
**BCLC stage**				
0	364 (4.9)	357 (4.9)	7 (5.6)	<0.001
A	3,006 (40.5)	2,929 (40.1)	77 (61.1)	
B	2,926 (39.4)	2,892 (39.6)	34 (27.0)	
C	1,132 (15.2)	1,124 (15.4)	8 (6.3)	
**Tumor thrombus**				
No	4,659 (63.4)	4,583 (63.2)	76 (79.2)	0.001
Yes	2,689 (36.6)	2,669 (36.8)	20 (20.8)	
**Tumor nodule**				
Single	5,498 (81.1)	5,422 (81.0)	76 (85.4)	0.293
Multiple	1,285 (18.9)	1,272 (19.0)	13 (14.6)	
**Tumor diameter (cm)**				
<3	1,034 (13.9)	1,008 (13.8)	26 (20.6)	0.027
≥3	6,422 (86.1)	6,322 (86.2)	100 (79.4)	
**AFP (ng/ml)**				
<20	2,897 (36.6)	2,820 (36.3)	77 (49.0)	0.001
≥20	5,027 (63.4)	4,947 (63.7)	80(51.0)	
**Tumor capsule**				
Yes	5,183 (76.3)	5,112 (76.3)	71(79.8)	0.439
No	1,609 (23.7)	1,591 (23.7)	18 (20.2)	
**Total bilirubin (µmol/L)**				
≤23	6,475 (88.9)	6,394 (89.0)	81 (84.4)	0.152
>23	806 (11.1)	791 (11.0)	15 (15.6)	
**Direct bilirubin (µmol/L)**				
≤8	5,768 (81.7)	5,714 (81.8)	54 (73.0)	0.051
>8	1,293 (18.3)	1,273 (18.2)	20 (27.0)	
**Albumin (g/L)**				
≥40	4,338 (61.1)	4,339 (61.2)	49 (51.6)	0.056
<40	2,795 (38.9)	2,749 (38.8)	46 (48.4)	

Data are shown in n (%).

### Risk Factors Related to the Overall Survival of Major Histotypes of PLC

In total, 7679 PLC patients who received curative surgery at the study medical centers were invited to participate in the follow-up study. Of those, 94 were excluded due to lack of survival data, and the remaining 7585 patients were included in the survival analysis. The median follow-up time was 1.50 years, with an IQR of 0.75–3.17 years. Of the 7585 PLC patients, 2809 died of this malignancy during follow-up, with the 1-, 3-, and 5-year survival rates of 79.7%, 56.9%, and 41.3%, respectively. The 1-, 3-, and 5-year survival rates were 80.7%, 58.3%, and 42.1% in HCC; 48.7%, 24.2%, and 18.5% in ICC; and 68.6%, 30.1%, and 23.8% in CHC. Of patients in the four regions, patients in the west (Wuwei, Gansu) were excluded from the analysis because they did not have qualified histological and prognostic data. The 1-, 3-, and 5-year survival rates of HCC patients were 74.6%, 42.3%, and 25.9% in Shanghai; 85.5%, 67.3%, and 49.9% in Nanning; and 92.5%, 83.6%, and 69.4% in Beijing, respectively. In order to analyze the relationship between BCLC stages and postoperative survival of PLC patients, the survival rate was calculated according to the different BCLC stages. We confirmed that the survival rate decreased significantly with increasing BCLC stages (*P*<0.001). Compared to HCC patients at BCLC B&C stage, HCC patients at 0&A stage had a better postoperative prognosis; the same was true for ICC patients (**Table 4**).

**Table 4:** Survival rate of HCC and ICC patients with different BCLC stage.

BCLC stage	Cases	1-year survival rate (%)	3-year survival rate (%)	5-year survival rate (%)	*P-*value
HCC					
0&A	3,343	92.4	77.2	64.8	<0.001^a^
B&C	3,697	69.8	40.5	29.2	
Subtotal	7,040	80.6	58.2	42.1	
ICC					
0&A	73	70.4	44.3	44.3	<0.001^a^
B&C	119	34.9	11.0	4.2	
Subtotal	192	48.7	24.2	18.5	

^a^ compared between 0&A stage and B&C stage.

The Cox proportional hazard model was applied to evaluate factors significantly associated with postoperative OS in HCC. The univariate Cox regression analysis identified 13 factors that were significantly associated with OS. The multivariate Cox regression analysis indicated that HBV seropositivity (HR, 1.29; 95% CI, 1.10-1.21), incomplete tumor capsule (1.58; 1.41-1.77), vascular tumor thrombus (2.12; 1.90-2.36), tumor diameter (≥3 cm) (1.65; 1.29-2.12), more advanced BCLC stage (2.11; 1.85-2.41), AFP (≥20ng/ml) (1.69; 1.50-1.91), and direct bilirubin (>8µmol/L) (1.27; 1.11-1.45) independently contributed to shorter OS in HCC. Post-operative RFA (0.65; 0.53-0.79) and second resection (0.43; 0.34-0.55) significantly improved OS in HCC (**Table 5**).

**Table 5:** Univariate and multivariate Cox regression analysis for risk factors of overall survival in HCC.

Variable		No. (%) of participants(n =7257)	Univariate analysis		Multivariate analysis	
			HR (95% CI)	*P*-value	HR (95% CI)	*P*-value
**Age**	<40	859 (11.8)	1			
		40–59	4,324 (59.6)	1.19 (1.05-1.36)	0.009		
	≥60	2,074 (28.6)	1.10 (1.00-1.20)	0.044		
**Gender**	Female	936 (12.9)	1				
		Male	6,321 (87.1)	1.19 (1.06-1.34)	0.004		
**HBV**	Negative	1,104 (15.2)	1			
	Positive	6,136 (84.8)	1.50 (1.33-1.69)	<0.001	1.29 (1.10-1.21)	0.002
**HCV**	Negative	4,801 (97.2)	1			
	Positive	138 (2.8)	0.59 (0.43-0.80)	0.001		
**Cirrhosis**	No	3,296 (48.8)	1			
	Yes	3,459 (51.2)	0.96 (0.89-1.04)	0.325		
**Ascites**	No	6,402 (95.8)	1			
	Yes	279 (4.2)	0.93 (0.77-1.13)	0.481		
**Tumor capsule**	Yes	4,169 (68.6)	1			
	No	1,906 (31.4)	1.32 (1.21-1.43)	<0.001	1.58 (1.41-1.77)	<0.001
**Tumor nodule**	Single	4,821 (78.6)	1			
	Multiple	1,314 (21.4)	1.23 (1.12-1.35)	<0.001		
**Tumor thrombus**	No	4,215 (65.5)	1			
	Yes	2,222 (34.5)	3.20 (2.95-3.47)	<0.001	2.12 (1.90-2.36)	<0.001
**BCLC stage**	0&A	3,343 (47.5)	1			
	B&C	3,697 (52.5)	3.37 (3.10-3.68)	<0.001	2.11 (1.85-2.41)	<0.001
**Tumor diameter (cm)**	<3	919 (13.0)	1			
	≥3	6,150 (87.0)	2.85 (2.43-3.34)	<0.001	1.65 (1.29-2.12)	<0.001
**AFP (ng/ml)**	<20	2,548 (36.1)	1			
	≥20	4,504 (63.9)	2.17 (1.98-2.37)	<0.001	1.69(1.50-1.91)	<0.001
**Total bilirubin (µmol/L)**	≤23	5,756 (86.6)	1			
	>23	889 (13.4)	0.97 (0.86-1.10)	0.656		
**Direct bilirubin (µmol/L)**	≤8	5,048 (79.7)	1			
	>8	1,285 (20.3)	1.19 (1.07-1.31)	0.001	1.27(1.11-1.45)	<0.001
**Albumin (g/L)**	≥40	3,537 (53.4)	1			
	<40	3,082 (46.6)	0.96 (0.88-1.03)	0.258		
**Post-operative TACE**	No	1,744 (44.1)	1			
	Yes	2,210 (55.9)	1.02 (0.92-1.12)	0.717		
**Post-operative RFA**	No	3,596 (92.0)	1			
	Yes	312 (8.0)	0.57 (0.48-0.69)	<0.001	0.65(0.53-0.79)	<0.001
**Reoperation**	No	3,580 (92.0)	1			
	Yes	310 (8.0)	0.39 (0.32-0.49)	<0.001	0.43(0.34-0.55)	<0.001
**Radiotherapy**	No	3,751 (96.3)	1			
	Yes	145 (3.7)	1.05 (0.84-1.31)	0.668		
**Chemotherapy**	No	3,787 (94.6)	1			
	Yes	218 (5.4)	0.85 (0.66-1.09)	0.201		
**Targeted therapy**	No	3,712 (95.6)	1			
	Yes	171 (4.4)	1.06 (0.87-1.30)	0.555		

In the survival analysis for ICC, the multivariate Cox regression analysis indicated that more advanced BCLC stage (1.72; 1.08-2.72) and AFP (≥20ng/ml) (1.58; 1.00-2.49) were independently associated with shorter OS, while reoperation (0.12; 0.02-0.89) was independently associated with longer OS in ICC (**Supplementary Table 3**).

### Postoperative Survival of HCC Patients Solely Caused by HBV or HCV Infection

**Figure 2:**
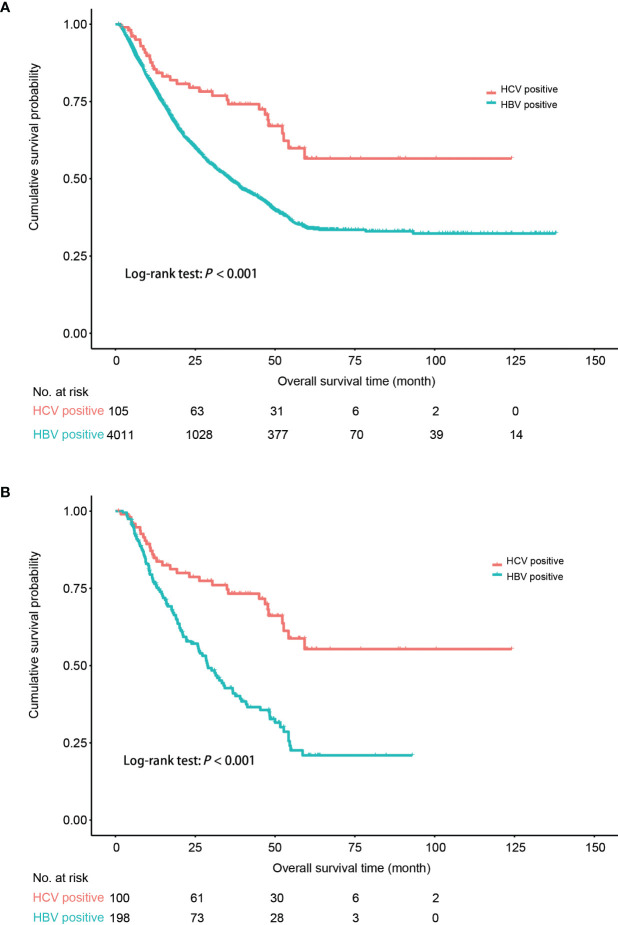
Comparison of postoperative survival probability between HCC patients solely infected with HBV and HCC patients solely infected with HCV. **(A)** All the patients. **(B)** Patients following the 1:2 propensity score (PS) matching with age, gender, and BCLC stage. HBV, hepatitis B virus; HCV, hepatitis C virus; HCC, hepatocellular carcinoma. Kaplan–Meier curves were plotted to visualize the difference.

As HBV seropositivity independently increased the risk of OS in PLC in the multivariate Cox analysis, we evaluated the effect of HBV and HCV infection on the prognosis of HCC. Log-rank test was applied to compare the difference in OS between HBV-HCC and HCV-HCC. The result indicated that HBV-HCC patients had an unfavorable prognosis compared to that of HCV-HCC (*P*<0.001) (**Figure 2A**). The PS matching with key baseline characteristics (age, gender, and BCLC stage) was applied to allow a common background for comparison between HBV-HCC and HCV-HCC, resulting in a matched sample size of 198 and 100, respectively. The result confirmed that HBV-HCC patients still had an unfavorable postoperative prognosis, compared to HCV-HCC patients (*P*<0.001) (**Figure 2B**).

### Prediction for Postoperative Prognosis in HCC

**Figure 3:**
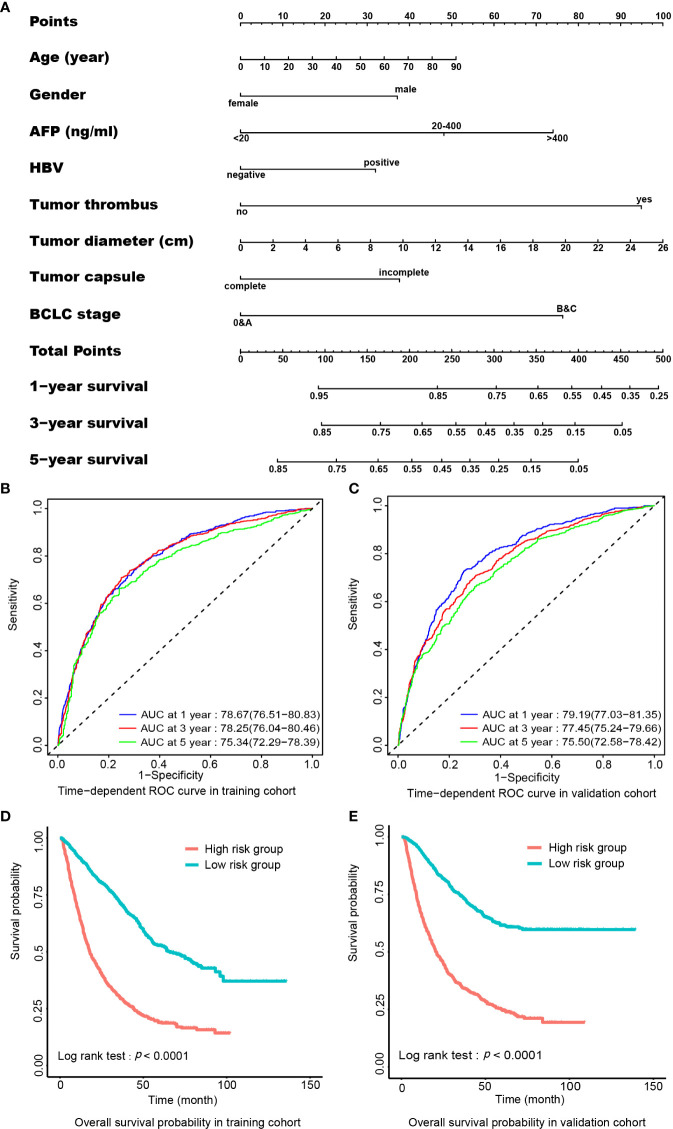
Preoperative nomogram for predicting postoperative survival in HCC. **(A)** The nomogram. To use this nomogram, a patient’s value is located on each variable axis, and a line represents the number of points received for each variable value. The sum of the score of each indicator is located on”Total Points” axis, and the total point represents the likelihood of postoperative survival of 1-, 3-, or 5-year shown on the survival axes. **(B)** AUC of time-dependent ROC curve for 1-, 3-, or 5-year survival in the training cohort. **(C)** AUC of time-dependent ROC curve for 1-, 3-, or 5-year survival in the validation cohort. **(D)** Comparison of OS probability between low- and high-risk groups according to total points from nomogram in the training cohort. **(E)** Comparison of overall survival probability between low- and high-risk group according to total points from nomogram in the validation cohort. AFP, α-fetoprotein; AUC, area under the curve; BCLC, Barcelona Clinic Liver Cancer; HBV, hepatitis B virus; HCC, hepatocellular carcinoma; OS, overall survival; ROC, receiver operating characteristics.

To evaluate if preoperative clinical parameters could predict postoperative prognosis of HCC, we developed a hazard risk prediction model consisting of independent preoperative prognostic factors. In the post-operative cohort of 7257 HCC patients, HCC patients were grouped randomly into a training cohort (*n*=3628) and a validation cohort (*n*=3629). All demographic and clinical characteristics were balanced between the two sub-cohorts (**Supplementary Table 4**). In the training cohort, the multivariate Cox analysis indicated that age, gender, incomplete tumor capsule, vascular tumor thrombus, HBV positivity, tumor diameter, AFP, and advanced BCLC stage were independently related to OS in HCC. A nomogram composed of these factors is shown in **Figure 3A**. The C-index for the prediction of survival in the training cohort and the validation cohort was 0.735 (95% CI, 0.727–0.743) and 0.733 (95% CI, 0.725–0.741), respectively. Time-dependent ROC was applied to evaluate the power of the prognosis prediction model formulated in this study. The result indicated that the area under the curve (AUC) was 0.79 (95% CI, 0.77-0.81) for 1-year survival, 0.78 (0.76-0.80) for 3-year survival, and 0.75 (0.72-0.78) for 5-year survival in the training cohort (**Figure 3B**). In the validation cohort, the AUC for 1-, 3-, and 5-year survival was 0.79 (0.77-0.81), 0.77 (0.75-0.80), and 0.76 (0.73-0.78), respectively (**Figure 3C**). According to the medium of the risk score calculated from the established hazard risk prediction model, the training cohort was classified into a high-risk group and a low-risk group. The survival analysis showed that the low-risk group had better OS probability than the high-risk group (*P*<0.001). This grouping method was verified also in the validation cohort, and the result was similar to that of the training cohort (**Figures 3D, E**).

The calibration plot was applied to evaluate the fitting degree of survival probability between the actual observation and prediction value calculated by the nomogram constructed in this study. The results displayed good fitness in the probability of 1-, 3-, and 5-year survivals both in the training cohort (**Supplementary Figures 3A, C, E**) and validation cohort (**Supplementary Figures 3B, D, F**).

## Discussion

In this study, we selected four medical centers distributed in the north, south, west, and east to represent the demographic, epidemiological, and clinical characteristics of PLC in China. The enrolled patients were mostly living in the provinces where study hospitals were located and near areas though they were from almost all provincial administrative regions of mainland China. The socioeconomic situations, living styles, and living environments of people from the four regions are mutually exclusive and different. The seropositivity of HBV in HCC patients was higher in the south and east than in the north and west. This is only partially coincident with the trend in their background HBV infection, as the prevalence of chronic HBV infection is lower in the north than in the south and east (21). The seropositivity of HBV in HCC in the west was lower than that in the east, although the prevalence of HBV was higher in the west than in the east (21). This is possibly because NAFLD, which is projected to become the leading cause of HCC in many countries (22), was more prevalent in the west (33.8%) than the other three regions (23). In addition, consumption of salted food containing N-nitroso compound and drinking water containing a high content of nitrate and nitrite were evident in Wuwei, Gansu, China (24). As the prevalence of PLC is closely related to geographic areas, socioeconomic state, and risk factor exposure, enrolled patients from the medical centers in the four regions should be highly representative among the current studies to characterize the overall risk factors, histotype composition, and prognostic factors in mainland China. We conclude that the seropositivity of HBV is 80.1% in PLC and 84.4% in HCC in mainland China; HCC, ICC, and CHC account for 93.0%, 4.3%, and 1.6% in PLC, respectively.

In this study, we found that the proportion of HCC in PLC increased consecutively from 89.70% before 2008 to 98.75% in 2016-2020, indicating the proportion of cholangiocarcinoma in PLC decreased correspondingly. Exposure to the risk factor of cholangiocarcinoma including liver fluke infection consecutively decreased. HBV seropositivity increased consecutively in PLC, but decreased in HCC, indicating that chronic HBV infection contributed increasingly to the occurrence of cholangiocarcinoma. It is possibly because HBV integration has been identified in 71.43% of ICCs (25). In this study, we found that the seropositivity of anti-HCV antibodies decreased consecutively, either in PLC or in HCC, although the incidence of HCV infection increased from 0.7 to 15.0 cases per 100,000 persons from 1997 to 2012 (26). This is possibly due to the fact that symptomatic hepatitis C has been treated in China over the past 20 years with medical insurance-covered interferon-α and ribavirin. In addition to interferon-α and ribavirin, direct-acting antivirals have fundamentally changed HCV-caused liver diseases, due to their high efficacy and tolerability (27, 28). HCC only derives from a diseased liver. HCV-induced carcinogenesis should be indirectly induced *via* multiple steps from chronic hepatitis to fibrosis, advanced fibrosis, and cirrhosis with somatic genetic/epigenetic alterations (27). In 1998, China enacted the blood donation law to strengthen the supervision of blood collection, organization, source management, and use of disposable syringes. Thereafter, the prevalence of hepatitis C in China decreased. These data support the observation of this study.

HBV infection led to an 8 year earlier onset in HCC, compared to HBV-free HCC. HBV-HCC patients had higher proportions of positive AFP, liver cirrhosis, advanced BCLC stage, multiple tumor nodules, and vascular tumor thrombus, indicating that HBV not only promotes the occurrence of HCC, but also promotes the recurrence of HCC. AFP can be upregulated by HBV X protein, which plays an important role in the aggressiveness of HCC by promoting HCC cells into stem cells and by activating the PI3K/mTOR signaling pathway (29). Liver cirrhosis is the result of an immune response to hepatic injury caused by chronic inflammation (30). HBV, especially its integrated forms in the human genome and its evolved forms generated in the long-term process of chronic infection, directly promotes the development of HCC (31–34). HBV replication, integration, and evolution also improve the recurrence and metastasis of HCC while long-term treatment of chronic HBV infection can reduce the development and postoperative recurrence of HBV-HCC (15, 35–39). As HCV-HCC is very rare in China, the large sample size in this study allows for identifying the difference in the clinical characteristics between HCV-HCC and HBV-HCC. Compared to HBV-HCC patients, HCV-HCC patients were 12 years older and had a lower proportion of some parameters indicating the aggressiveness and metastasis of HCC. As the BCLC stage is the major prognostic factor in HCC, the PS matching with age, gender, and BCLC stage was applied to allow a common background for comparison. The prognosis of HCV-HCC was also proven to be significantly better than that of HBV-HCC. The mechanism of HBV- and HCV-induced hepatocarcinogenesis should be different. HCV itself might not be directly oncogenic. Chronic HCV infection causes hepatic inflammation, necrosis, metabolic disorders, steatosis, regeneration, and cirrhosis, thus facilitating the development of HCC by creating an immunosuppressive tumorigenic environment and activating cancer stem-like cells by proinflammatory factors like plasminogen activator inhibitor-1 (40, 41). Other non-B and non-C risk factors including diabetes and NAFLD might also facilitate the development of HCC, mostly in elderly patients, and the overall survival rate is significantly better than that of HBV-HCC (42), possibly by inducing systemic and hepatic inflammation. Thus, HBV is directly carcinogenic. HBV replication, viral mutation, and integration into the host genome promote the development and progression of HCC. The non-HBV etiological factors including HCV infection promote the development of HCC mostly by inducing non-resolving inflammation which leads to the development of tumors by promoting proliferative and survival signaling, inducing instability of genome, and subsequent angiogenesis. Of note, non-resolving inflammation is also an important factor for the development and progression of HBV-HCC. These data clearly indicate that HBV is more carcinogenic than HCV orany other cause. Antiviral treatment is effective in decreasing the occurrence and postoperative recurrence of HCC in HBV-infected patients (15, 43). Thus, the prophylactic and therapeutic effects of anti-HBV treatment on the development of HCC should be added into the current clinical guidelines.

In this study, we showed that the overall 1 -, 3 -, and 5-year OS rates of HCC patients were 80.7%, 58.3%, and 42.1%, respectively. Importantly, the survival rates decrease with increasing BCLC stages (**Table 4**). The 5-year OS rate of HCC patients in the early BCLC stage (stage 0 and A) was 64.8%. It has been shown that the 5-year survival rate of HCC patients at early BCLC (stage 0/A) is 60% to 90% (44–47). The 5-year OS rates of patients enrolled in Shanghai were lower than those in Nanning and Beijing, possibly because the BCLC stage of HCC patients enrolled in Shanghai was more advanced than those enrolled in the other two cities (**Table 1**). Shanghai is usually the last station of the medical tour for patients to seek the best treatments because Shanghai has the top medical facilities in HCC surgery. Surprisingly, we found that the overall 1 -, 3 -, and 5-year OS rates increased consecutively in HCC, which is consistent with the increasing proportions of early-stage HCC (**Figure 1**). The outcomes of this study strongly suggest the necessity of timely screening for HCC in the high-risk population, especially the high-risk HBV-infected subjects who carry a high viral load and HCC-risk HBV mutations (15, 34–36), to increase the detection of HCC at an early stage.

In this study, we demonstrated that HBV seropositivity, AFP, incomplete tumor capsule, tumor diameter, advanced BCLC stage, and vascular tumor thrombus were independently associated with an unfavorable prognosis in HCC by the multivariate regression analysis. These factors are measurable before surgery. We also developed a nomogram composed of these preoperative prognostic factors and confirmed that this nomogram was able to accurately predict an unfavorable postoperative prognosis in HCC, even for the 5-year OS rate (**Figure 3**). The high accuracy of the nomogram established by preoperative parameters of the large sample size for the prediction of postoperative prognosis suggests that this nomogram should be extremely important for clinical decision-making. Predictors in our prognosis-prediction nomogram differ a little from the current predictors in HCC. The predictors of reported nomograms for HCC prognosis were mostly extracted after surgical treatment, including MVI and resection margin (48, 49). Development of a nomogram to preoperatively estimate postoperative survival is also reported (50). However, the predictor is different from our study. We identified predictors from presurgical parameters including HBV seropositivity to determine if patients were suitable for surgical treatment. The Shanghai score, one of the clinical stages of liver cancer that used HBV information as predictors, has 14 predictors (51). Our nomogram has eight predictors that are easy to be used clinically. We also confirmed that post-operative RFA and reoperation after recurrence independently increased OS in HCC (**Table 5**). Thus, post-operative RFA and reoperation after recurrence should be options to improve the therapeutic regimen.

In this study, we also found that HBV infection was associated with 5-year earlier onset and higher AFP, liver cirrhosis, advanced BCLC stage, and vascular tumor thrombus in ICC. Published studies indicated that antiviral treatment decreased the risk and prolonged long-term survival in ICC (52, 53). This line of evidence reflects the role of HBV in generating inflammatory background from which ICC develops. In addition, HBV integration is frequently identified in ICC and other cancer types including non-Hodgkin lymphoma (24, 54). Significant associations of HBV seropositivity with leukemia, extrahepatic bile duct carcinoma, esophageal cancer, stomach cancer, and pancreatic cancer are also suggested (55). We hypothesize that weak antiviral and anti-cancer immunity predisposed by genetic and environmental exposure arouse cancer-promoting non-resolving inflammation, which facilitates the development of ICC and extrahepatic cancers.

There are several limitations in our study. First, the relatively weak risk factors of PLC, namely NAFLD, diabetes, nonalcoholic steatohepatitis, alcoholic liver disease, aflatoxin exposure, liver fluke exposure, family history, cigarette smoking, and alcohol consumption (5–7, 56) were not included because these data were incomplete in medical records. The contributions of these risk factors to HBV- or HCV-related HCC remain unknown. Second, clinical data related to curative surgery, pathological examination, and follow-up in Wuwei (the west) did not meet the criteria, and were therefore not included in the analysis, resulting in a loss of data. Third, compared to patients who did not join the follow-up study, patients who were followed up had a higher proportion of HBV positivity, high AFP (≥20ng/ml), poor liver function, multiple tumor nodules, incomplete tumor capsules, and late BCLC stage (**Supplementary Table 5**). These factors were mostly associated with an unfavorable prognosis in HCC. The postoperative survival might be underestimated. Fourth, ICC and CHC were not analyzed for prognosis prediction because of small sample sizes.

Conclusively, chronic HBV infection contributes to 84.4% of HCC in mainland China. HBV infection not only induces hepatocarcinogenesis but also promotes the aggressiveness of HCC. HCV-HCC onset is 12 years later than HBV-HCC and has a better prognosis than HBV-HCC. A significant postoperative survival benefit is obtained in patients at the early BCLC stage, highlighting the importance of screening for early-stage HCC in high-risk populations. Our prognosis prediction model constructed with preoperational parameters is important for clinical decision making.

## Data Availability Statement

The raw data supporting the conclusions of this article will be made available by the authors, without undue reservation.

## Ethics Statement

The studies involving human participants were reviewed and approved by Second Military Medical University. Written informed consent to participate in this study was provided by the participants’ legal guardian/next of kin.

## Author Contributions

GC contributed to study design and supervision. HY, XB, WZ, WT, JC, and ZS contributed to patient enrolment, acquisition of data, and follow-up. HZ, JL, JY, MH, PZ, and JW conducted data organization, statistical analysis, and data interpretation. XL, CQ, MW, KL, YW, and ZZ contributed to data collection and follow-up. GC wrote the manuscript. All authors had access to the data and approved the final version of this manuscript.

## Funding

This study was supported by the National Key Basic Research Program of China (973 Program) (grant numbers 2015CB554000 to GC), Key discipline from the “3-year public health promotion” program of Shanghai Municipal Health Commission to GC (GWV-10.1-XK17), the State Key Infection Disease Project of China (grant numbers 2017ZX10201201-006-001 to HZ), the National Natural Science Foundation of China (grant numbers 81520108021, 91529305,and 81673250 to GC), the Key Research and Development Project of Guangxi (grant number AB18050020 to HY and grant number AA18221001 to WT), the Natural Science Foundation of Guangxi (grant number 2018GXNSFDA050012 to HY), and Shanghai Wu Mengchao Medical Science Foundation (grant number JJHXM-2019042 to HY).

## Conflict of Interest

The authors declare that the research was conducted in the absence of any commercial or financial relationships that could be construed as a potential conflict of interest.

## Publisher’s Note

All claims expressed in this article are solely those of the authors and do not necessarily represent those of their affiliated organizations, or those of the publisher, the editors and the reviewers. Any product that may be evaluated in this article, or claim that may be made by its manufacturer, is not guaranteed or endorsed by the publisher.

## Supplementary Material

Supplementary Figure 1 Diagram of patients enrolled in this study.

Supplementary Figure 2 The geographic distribution of the patients with primary liver cancer in the study.

Supplementary Figure 3 The calibration curve for predicting postoperative survival with preoperative nomogram in HCC. **(A)** The calibration curve for predicting postoperative 1-year survival in the training cohort. **(B)** The calibration curve for predicting postoperative 1-year survival in the validation cohort. **(C)** The calibration curve for predicting postoperative 3-year survival in the training cohort. **(D)** The calibration curve for predicting postoperative 3-year survival in the validation cohort. **(E)** The calibration curve for predicting postoperative 5-year survival in the training cohort. **(F)** The calibration curve for predicting postoperative 5-year survival in the validation cohort.
