# Supplementary figures and images for: Epidemiological Characteristics of Primary Liver Cancer in Mainland China From 2003 to 2020: A Representative Multicenter Study
Source: Front Oncol. 2022 Jun 21;12:906778. doi: 10.3389/fonc.2022.906778 (PMC9253580; doi:10.3389/fonc.2022.906778)

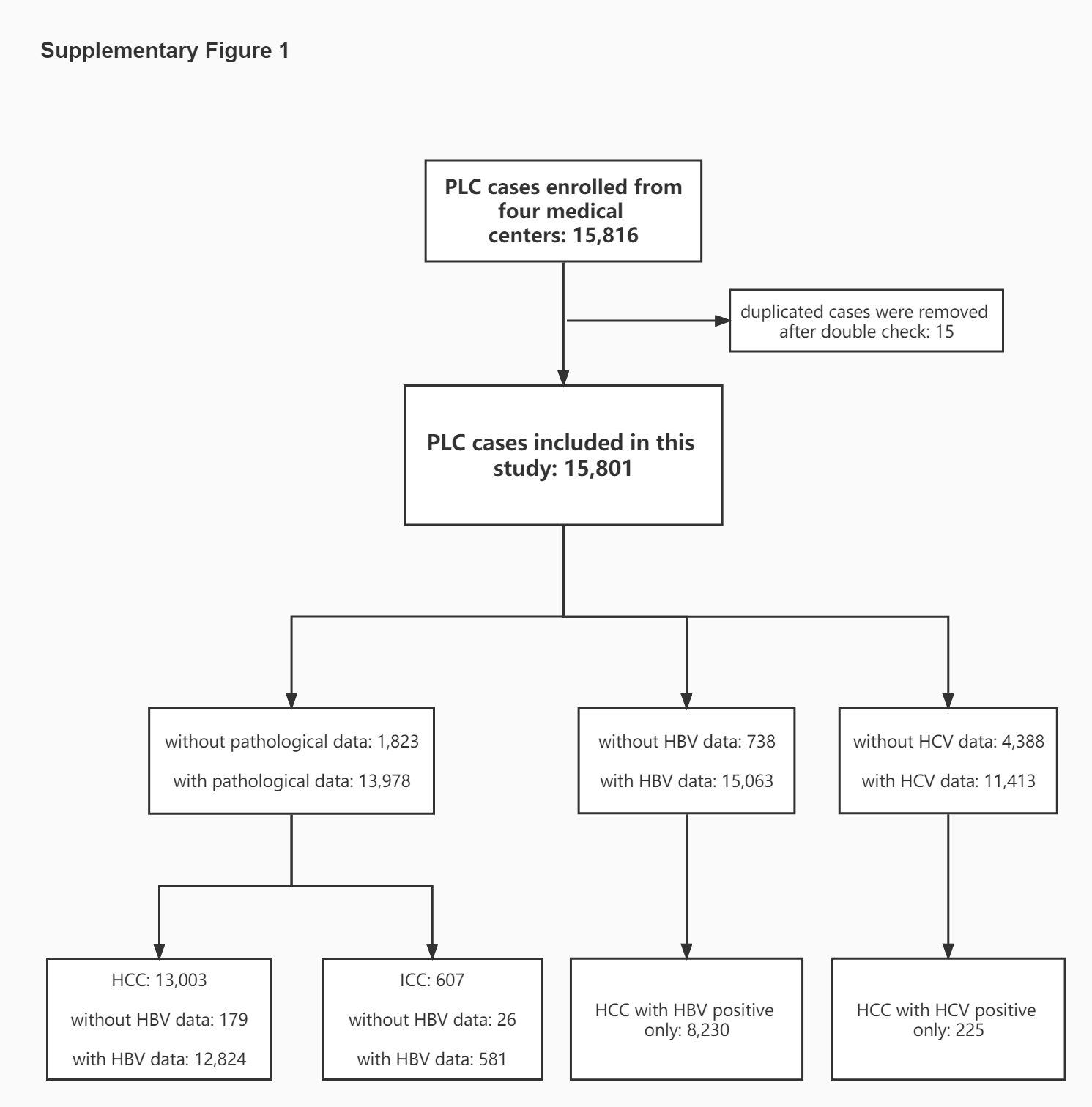

Supplement: Supplementary Figure 1 — Diagram of patients enrolled in this study. [file Image_1.jpeg]

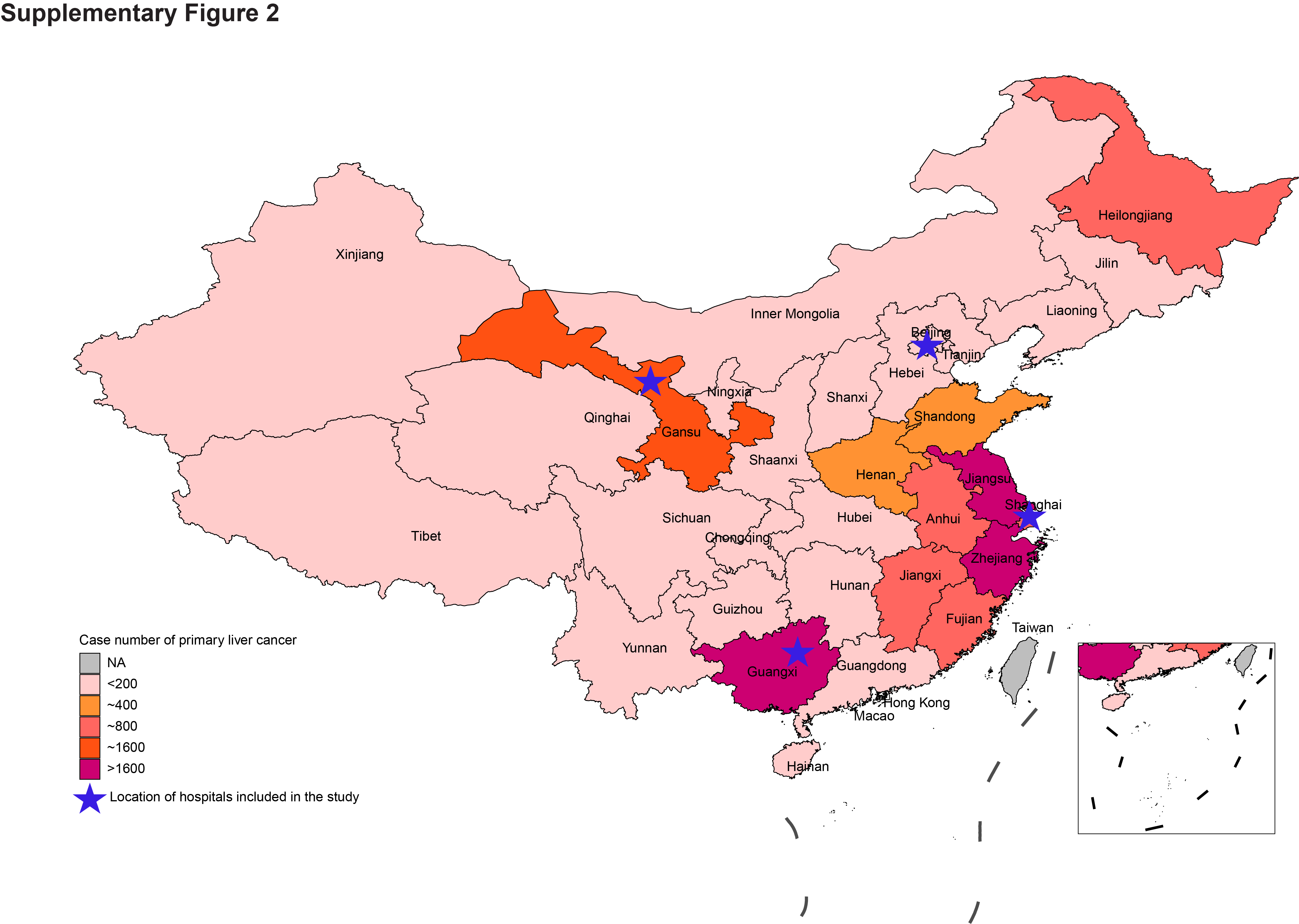

Supplement: Supplementary Figure 2 — The geographic distribution of the patients with primary liver cancer in the study. [file Image_2.jpeg]

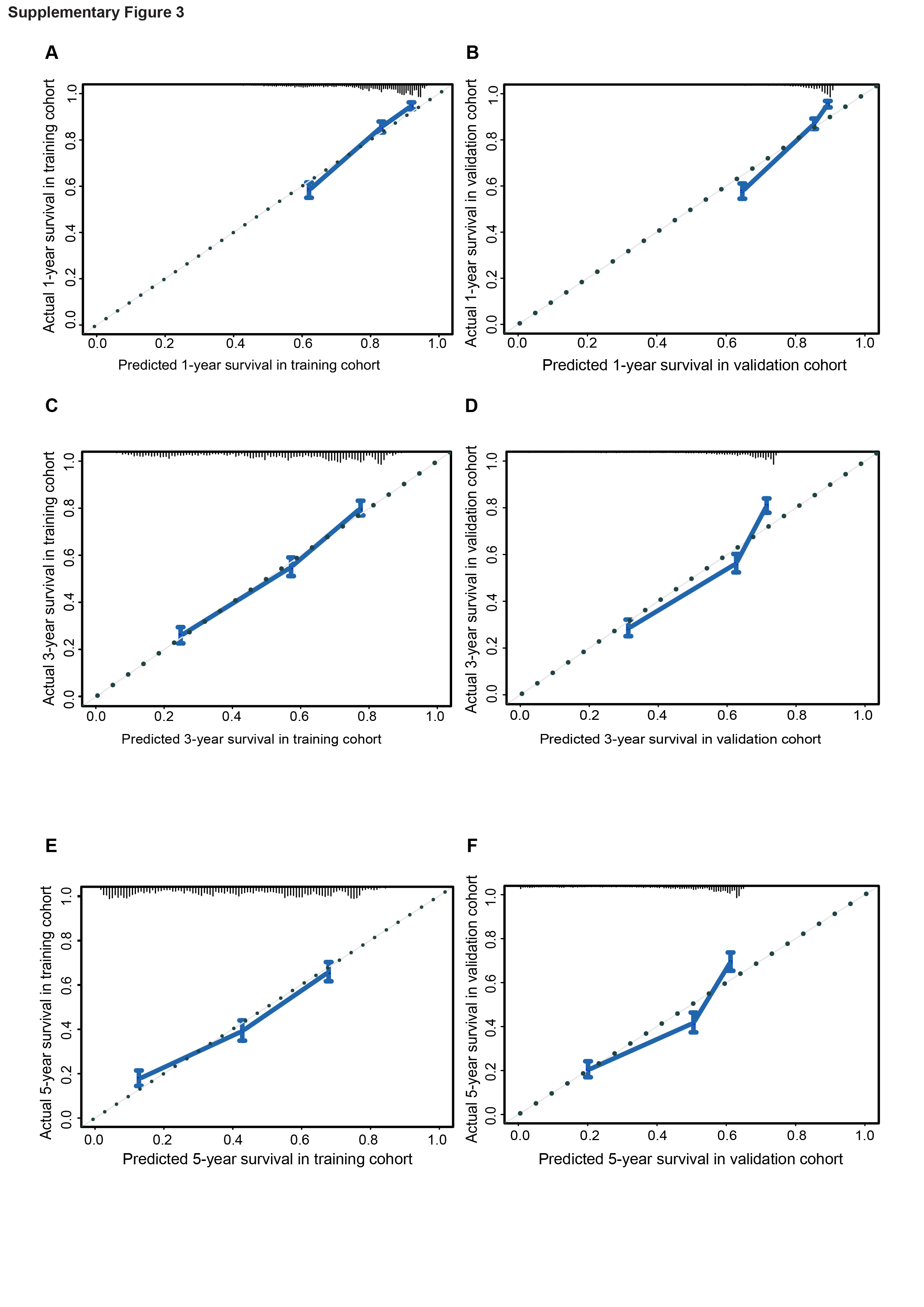

Supplement: Supplementary Figure 3 — The calibration curve for predicting postoperative survival with preoperative nomogram in HCC. (A) The calibration curve for predicting postoperative 1-year survival in the training cohort. (B) The calibration curve for predicting postoperative 1-year survival in the validation cohort. (C) The calibration curve for predicting postoperative 3-year survival in the training cohort. (D) The calibration curve for predicting postoperative 3-year survival in the validation cohort. (E) The calibration curve for predicting postoperative 5-year survival in the training cohort. (F) The calibration curve for predicting postoperative 5-year survival in the validation cohort. [file Image_3.jpeg]
