# Supplementary material for: Epidemiological Characteristics of Primary Liver Cancer in Mainland China From 2003 to 2020: A Representative Multicenter Study
Source: Front Oncol. 2022 Jun 21;12:906778. doi: 10.3389/fonc.2022.906778 (PMC9253580; doi:10.3389/fonc.2022.906778)
Supplement: Supplementary file 4 [file Table_1.docx]

**Supplementary Table 1.** Demographic and clinical characteristics of PLC patients from four medical centers in China

| **Variable** | **Total**  (n=15801) | **Female**  (n=2519) | **Male**  (n=13282) | ***P* value** |
| --- | --- | --- | --- | --- |
| **Age (yr)** |  |  |  |  |
| Medium (IQR) | 54(46-62) | 56(48-63) | 54(46-62) |  |
| <40 | 1,562(9.9) | 224(8.9) | 1,338(10.1) | <0.001 |
| 40–59 | 9,098(57.6) | 1,335(53.0) | 7,763(58.4) |  |
| ≥60 | 5,141(32.6) | 960(38.1) | 4,181(31.5) |  |
| **HBV** |  |  |  |  |
| Negative | 2,999(19.9) | 670(29.0) | 2,329(18.3) | <0.001 |
| Positive | 12,064(80.1) | 1,637(71.0) | 10,427(81.7) |  |
| **HCV** |  |  |  |  |
| Negative | 11,027(96.6) | 1,658(95.6) | 9,369(96.8) | 0.008 |
| Positive | 386(3.4) | 77(4.4) | 309(3.2) |  |
| **AFP (ng/ml)** |  |  |  |  |
| <20 | 4,940(39.3) | 565(32.5) | 4,375(40.4) | <0.001 |
| ≥20 | 7,618(60.7) | 1,171(67.5) | 6,447(59.6) |  |
| **Albumin (g/L)** |  |  |  |  |
| ≥40 | 6,661(59.6) | 878(57.2) | 5,783(60.0) | 0.037 |
| <40 | 4,518(40.4) | 658(42.8) | 3,860(40.0) |  |
| **Total bilirubin (µmol/L)** |  |  |  |  |
| ≤23 | 10,077(88.9) | 1,442(92.4) | 8,635(88.3) | <0.001 |
| >23 | 1,257(11.1) | 118(7.6) | 1,139(11.7) |  |
| **Direct bilirubin (µmol/L)** |  |  |  |  |
| ≤8 | 8,976(81.5) | 1,342(88.6) | 7,634(80.4) | <0.001 |
| >8 | 2,035(18.5) | 172(11.4) | 1,863(19.6) |  |
| **Cirrhosis** |  |  |  |  |
| No | 6,272(49.8) | 941(53.0) | 5,331(49.3) | 0.004 |
| Yes | 6,318(50.2) | 834(47.0) | 5,484(50.7) |  |
| **Ascites** |  |  |  |  |
| No | 11,205(96.7) | 1,558(96.1) | 9,647(96.8) | 0.145 |
| Yes | 381(3.3) | 63(3.9) | 318(3.2) |  |
| **BCLC stage** |  |  |  |  |
| 0 | 472(4.0) | 84(5.1) | 388(3.8) | 0.001 |
| A | 4,906(41.6) | 729(44.4) | 4,177(41.2) |  |
| B | 4,330(36.7) | 584(35.5) | 3,746(36.9) |  |
| C | 2,076(17.6) | 246(15.0) | 1,830(18.0) |  |
| **Pathological type** |  |  |  |  |
| HCC | 13,003(93.0) | 1,755(85.7) | 11,248(94.3) | <0.001 |
| ICC | 607 (4.3) | 216(10.5) | 391 (3.3) |  |
| CHC | 222 (1.6) | 41 (2.0) | 181 (1.5) |  |
| Others | 146 (1.0) | 36 (1.8) | 110 (0.9) |  |

Data are shown in n (%).
