# Supplementary material for: Epidemiological Characteristics of Primary Liver Cancer in Mainland China From 2003 to 2020: A Representative Multicenter Study
Source: Front Oncol. 2022 Jun 21;12:906778. doi: 10.3389/fonc.2022.906778 (PMC9253580; doi:10.3389/fonc.2022.906778)
Supplement: Supplementary file 5 [file Table_2.docx]

**Supplementary Table 2.** Comparison of demographic and clinical characteristics between ICC patients with and without HBV infection

| **Variable** | **Total**  **(n=581)** | **HBV Negative**  **(n=357)** | **HBV Positive**  **(n=224)** | ***P* value** |
| --- | --- | --- | --- | --- |
| **Age（years）** |  |  |  |  |
| Medium (IQR) | 57(49-64) | 59(51-66) | 54 (48-61) | <0.001 |
| <40 | 30 (5.2) | 21 (5.9) | 9 (4.0) |  |
| 40–59 | 316(54.4) | 165(46.2) | 151(67.4) |  |
| ≥60 | 235(40.4) | 171(47.9) | 64(28.6) |  |
| **Gender** |  |  |  |  |
| Female | 203(34.9) | 155(43.4) | 48(21.4) | <0.001 |
| Male | 379(65.1) | 202(56.6) | 176(78.6) |  |
| **AFP (ng/ml)** |  |  |  |  |
| <20 | 305(80.7) | 183(92.0) | 122(68.2) | <0.001 |
| ≥20 | 73(19.3) | 16 (8.0) | 57(31.8) |  |
| **HCV** |  |  |  |  |
| Negative | 468(98.3) | 290(98.0) | 178(98.9) | 0.451 |
| Positive | 8 (1.7) | 6 (2.0) | 2(1.1) |  |
| **Cirrhosis** |  |  |  |  |
| No | 253(72.9) | 154(89.0) | 99(56.9) | <0.001 |
| Yes | 94(27.1) | 19(11.0) | 75(43.1) |  |
| **BCLC stage** |  |  |  |  |
| 0 | 4(1.2) | 2(1.2) | 2(1.2) | 0.010 |
| A | 107(32.2) | 67(40.8) | 40(23.8) |  |
| B | 157(47.3) | 69(42.1) | 88(52.4) |  |
| C | 64(19.3) | 26(15.9) | 38(22.6) |  |
| **Tumor thrombus** |  |  |  |  |
| No | 223(77.2) | 104(83.2) | 119(72.6) | 0.033 |
| Yes | 66(22.8) | 21(16.8) | 45(27.4) |  |
| **Tumor nodule** |  |  |  |  |
| Single | 241(82.8) | 109(85.2) | 132(81.0) | 0.349 |
| Multiple | 50(17.2) | 19(14.8) | 31(19.0) |  |
| **Tumor diameter (cm)** |  |  |  |  |
| <3 | 21(6.4) | 11(6.7) | 10(6.0) | 0.788 |
| ≥3 | 308(93.6) | 152(93.3) | 156(94.0) |  |
| **Tumor capsule** |  |  |  |  |
| Yes | 47(16.3) | 13(10.4) | 34(20.9) | 0.017 |
| No | 241(83.7) | 112(89.6) | 129(79.1) |  |
| **Total bilirubin (µmol/L)** |  |  |  |  |
| ≤23 | 273(92.9) | 122(94.6) | 151(91.5) | 0.312 |
| >23 | 21(7.1) | 7(5.4) | 14(8.5) |  |
| **Direct bilirubin (µmol/L)** |  |  |  |  |
| ≤8 | 246(86.3) | 112(90.3) | 134(83.2) | 0.084 |
| >8 | 39(13.7) | 12(9.7) | 27(16.8) |  |
| **Albumin (g/L)** |  |  |  |  |
| ≥40 | 224(78.6) | 107(83.6) | 117(74.5) | 0.063 |
| <40 | 61(21.4) | 21(16.4) | 40(25.5) |  |

Data are shown in n (%).
