# Supplementary material for: Epidemiological Characteristics of Primary Liver Cancer in Mainland China From 2003 to 2020: A Representative Multicenter Study
Source: Front Oncol. 2022 Jun 21;12:906778. doi: 10.3389/fonc.2022.906778 (PMC9253580; doi:10.3389/fonc.2022.906778)
Supplement: Supplementary file 6 [file Table_3.docx]

**Supplementary Table 3.** Univariate and multivariate Cox regression analysis of risk factors for overall survival in ICC

| **Variable** | | **No.(%) of participants**  **(n =191)** | **Univariate analysis** | |  | **Multivariate analysis** | |
| --- | --- | --- | --- | --- | --- | --- | --- |
|  |  |  | **HR(95% CI)** | ***P* value** |  | **HR (95% CI)** | ***P* value** |
| **Age** | <40 | 5(2.6) | 1 |  |  |  |  |
|  | 40–59 | 104(54.5) | 0.87(0.27-2.79) | 0.653 |  |  |  |
|  | ≥60 | 82(42.9) | 1.13(0.80-1.58) | 0.492 |  |  |  |
| **Gender** | Female | 73(38.2) | 1 |  |  |  |  |
|  | Male | 118(61.8) | 1.32(0.93-1.86) | 0.118 |  |  |  |
| **Cirrhosis** | No | 143(75.7) | 1 |  |  |  |  |
|  | Yes | 46(24.3) | 0.73(0.49-1.09) | 0.127 |  |  |  |
| **HBV** | Negative | 117(61.3) | 1 |  |  |  |  |
|  | Positive | 74(38.7) | 1.97(1.40-2.76) | <0.001 |  |  |  |
| **HCV** | Negative | 157(98.1) | 1 |  |  |  |  |
|  | Positive | 3(1.9) | 0.59(0.15-2.39) | 0.458 |  |  |  |
| **Tumor capsule** | Yes | 12(8.2) | 1 |  |  |  |  |
|  | No | 135(91.8) | 1.33(0.68-2.63) | 0.408 |  |  |  |
| **Tumor nodule** | Single | 121(81.8) | 1 |  |  |  |  |
|  | Multiple | 27(18.2) | 1.59(1.01-2.50) | 0.044 |  |  |  |
| **Tumor thrombus** | No | 117(79.6) | 1 |  |  |  |  |
|  | Yes | 30(20.4) | 1.53(0.98-2.40) | 0.064 |  |  |  |
| **BCLC stage** | 0&A | 72(37.9) | 1 |  |  |  |  |
|  | B&C | 118(62.1) | 2.83(1.94-4.12) | <0.001 |  | 1.72(1.08-2.72) | 0.022 |
| **Tumor diameter (cm)** | <3 | 11(5.9) | 1 |  |  |  |  |
|  | ≥3 | 177(94.1) | 1.36(0.66-2.77) | 0.404 |  |  |  |
| **AFP (ng/ml)** | <20 | 151(81.2) | 1 |  |  |  |  |
|  | ≥20 | 35(18.8) | 1.89(1.24-2.90) | 0.003 |  | 1.58(1.00-2.49) | 0.050 |
| **Total bilirubin (µmol/L)** | ≤23 | 136(91.9) | 1 |  |  |  |  |
|  | >23 | 12(8.1) | 0.94(0.49-1.79) | 0.842 |  |  |  |
| **Direct bilirubin (µmol/L)** | ≤8 | 126(85.1) | 1 |  |  |  |  |
|  | >8 | 22(14.9) | 1.02(0.63-1.67) | 0.932 |  |  |  |
| **Albumin (g/L)** | ≥40 | 121(82.9) | 1 |  |  |  |  |
|  | <40 | 25(17.1) | 1.59(1.01-2.52) | 0.046 |  |  |  |
| **Post-operative TACE** | No | 102(65.0) | 1 |  |  |  |  |
|  | Yes | 55(35.0) | 0.62(0.43-0.90) | 0.011 |  |  |  |
| **Post-operative RFA** | No | 145(94.8) | 1 |  |  |  |  |
|  | Yes | 8(5.2) | 0.52(0.23-1.19) | 0.120 |  |  |  |
| **Reoperation** | No | 144(94.1) | 1 |  |  |  |  |
|  | Yes | 9(5.9) | 0.19(0.06-0.59) | 0.004 |  | 0.12(0.02-0.89) | 0.038 |
| **Radiotherapy** | No | 132(86.3) | 1 |  |  |  |  |
|  | Yes | 21(13.7) | 0.97(0.59-1.57) | 0.886 |  |  |  |
| **Chemotherapy** | No | 144(94.7) | 1 |  |  |  |  |
|  | Yes | 8 (5.3) | 1.21(0.53-2.75) | 0.652 |  |  |  |
