# Supplementary material for: Epidemiological Characteristics of Primary Liver Cancer in Mainland China From 2003 to 2020: A Representative Multicenter Study
Source: Front Oncol. 2022 Jun 21;12:906778. doi: 10.3389/fonc.2022.906778 (PMC9253580; doi:10.3389/fonc.2022.906778)
Supplement: Supplementary file 7 [file Table_4.docx]

**Supplementary Table 4.** Comparison of the characters between sub-cohorts of training and validation

| **Variable** | **Training cohort**  **(n=3,628)** | **Validation cohort**  **(n=3,629)** | ***P* value** |
| --- | --- | --- | --- |
| **Gender** |  |  |  |
| Female | 447 (12.3) | 489 (13.5) | 0.152 |
| Male | 3,181 (87.7) | 3,140 (86.5) |  |
| **Age** |  |  |  |
| Medium (IQR) | 52(45~61) | 53(45~60) | 0.930 |
| <40 | 434 (12.0) | 425 (11.7) | 0.806 |
| 40–59 | 2,148 (59.2) | 2,176 (60.0) |  |
| ≥60 | 1,046 (28.8) | 1,028 (28.3) |  |
| **Cirrhosis** |  |  |  |
| No | 1,664 (49.3) | 1,632 (48.3) | 0.429 |
| Yes | 1,712 (50.7) | 1,747 (51.7) |  |
| **Ascites** |  |  |  |
| No | 3,177 (95.5) | 3,225 (96.2) | 0.196 |
| Yes | 150 (4.5) | 129 (3.8) |  |
| **HBV** |  |  |  |
| Negative | 574 (15.9) | 530 (14.6) | 0.160 |
| Positive | 3,046 (84.1) | 3,090 (85.4) |  |
| **HCV** |  |  |  |
| Negative | 2,381 (97.0) | 2,420 (97.4) | 0.397 |
| Positive | 74 (3.0) | 64 (2.6) |  |
| **Tumor thrombus** |  |  |  |
| No | 2,117 (66.0) | 2,098 (65.0) | 0.445 |
| Yes | 1,093 (34.0) | 1,129 (35.0) |  |
| **Tumor diameter (cm)** |  |  |  |
| <3 | 449 (12.7) | 470 (13.3) | 0.460 |
| ≥3 | 3,089 (87.3) | 3,061 (86.7) |  |
| **Tumor nodule** |  |  |  |
| Single | 2,398 (78.3) | 2,423 (78.9) | 0.598 |
| Multiple | 665 (21.7) | 649 (21.1) |  |
| **Tumor capsule** |  |  |  |
| No | 2,074 (68.5) | 2,095 (68.8) | 0.847 |
| Yes | 954 (31.5) | 952 (31.2) |  |
| **AFP (ng/ml)** |  |  |  |
| <20 | 1,282 (36.4) | 1,268 (35.9) | 0.215 |
| 20-400 | 890 (25.3) | 842 (23.9) |  |
| >400 | 1,351 (38.3) | 1,419 (40.2) |  |
| **Direct bilirubin (µmol/L)** |  |  |  |
| ≤8 | 2,505 (79.7) | 2,543 (79.7) | 0.972 |
| >8 | 639 (20.3) | 646 (20.3) |  |
| **Albumin (g/L)** |  |  |  |
| ≥40 | 1,749 (53.0) | 1,788 (53.8) | 0.526 |
| <40 | 1,549 (47.0) | 1,533 (46.2) |  |
| **BCLC stage** |  |  |  |
| 0&A | 1,652 (46.9) | 1,691 (48.0) | 0.364 |
| B&C | 1,868 (53.1) | 1,829 (52.0) |  |

Data are shown in n (%).
