# Supplementary material for: Epidemiological Characteristics of Primary Liver Cancer in Mainland China From 2003 to 2020: A Representative Multicenter Study
Source: Front Oncol. 2022 Jun 21;12:906778. doi: 10.3389/fonc.2022.906778 (PMC9253580; doi:10.3389/fonc.2022.906778)
Supplement: Supplementary file 8 [file Table_5.docx]

**Supplementary Table 5.** Demographic and clinical characteristics between PLC patients in follow-up and without

| **Variable** | **Total**  (n=15,801) | **Follow-up**  (n=7,679) | **No follow-up**  (n=8,122) | ***P* value** |
| --- | --- | --- | --- | --- |
| **Age (yr)** |  |  |  |  |
| Medium (IQR) | 54(46-62) | 53(45-61) | 55(47-63) | <0.001 |
| <40 | 1,562 (9.9) | 887 (11.6) | 675(8.3) |  |
| 40–59 | 9,098 (57.6) | 4,554 (59.3) | 4,544(55.9) |  |
| ≥60 | 5,141 (32.5) | 2,238 (29.1) | 2,903(35.7) |  |
| **Gender** |  |  |  |  |
| Male | 2,519 (15.9) | 1,048 (13.6) | 1,471(18.1) | <0.001 |
| Female | 13,282 (84.1) | 6,631 (86.4) | 6,651(81.9) |  |
| **HBV** |  |  |  |  |
| Negative | 2,999 (19.9) | 1,267 (16.5) | 1,732(23.4) | <0.001 |
| Positive | 12,064 (80.1) | 6,391 (83.5) | 5,673(76.6) |  |
| **HCV** |  |  |  |  |
| Negative | 11,027 (96.6) | 5,132 (97.2) | 5,895(96.1) | 0.001 |
| Positive | 386 (3.4) | 148 (2.8) | 238(3.9) |  |
| **AFP (ng/ml)** |  |  |  |  |
| <20 | 4,940 (39.3) | 2,779 (37.3) | 2,161(42.3) | <0.001 |
| ≥20 | 7,618 (60.7) | 4,672 (62.7) | 2,946(57.7) |  |
| **Albumin (g/L)** |  |  |  |  |
| ≥40 | 6,661 (59.6) | 3,761 (53.9) | 2,900(69.0) | <0.001 |
| <40 | 4,518 (40.4) | 3,215 (46.1) | 1,303(31.0) |  |
| **Total bilirubin (µmol/L)** |  |  |  |  |
| ≤23 | 10,077 (88.9) | 6,057 (86.5) | 4,020(92.8) | <0.001 |
| >23 | 1,257 (11.1) | 947 (13.5) | 310(7.2) |  |
| **Direct bilirubin (µmol/L)** |  |  |  |  |
| ≤8 | 8,976 (81.5) | 5,298 (79.8) | 3,678(84.2) | <0.001 |
| >8 | 2,035 (18.5) | 1,345 (20.2) | 690(15.8) |  |
| **Cirrhosis** |  |  |  |  |
| No | 6,272 (49.8) | 3,528 (49.6) | 2,744(50.1) | 0.529 |
| Yes | 6,318 (50.2) | 3,589 (50.4) | 2,729(49.9) |  |
| **Ascites** |  |  |  |  |
| No | 11,205 (96.7) | 6,760 (95.9) | 4,445(97.9) | <0.001 |
| Yes | 381 (3.3) | 287 (4.1) | 94(2.1) |  |
| **BCLC stage** |  |  |  |  |
| 0 | 472 (4.0) | 260 (3.5) | 212(4.9) | <0.001 |
| A | 4,906 (41.6) | 3,263 (43.8) | 1,643(37.9) |  |
| B | 4,330 (36.7) | 2,469 (33.1) | 1,861(43.0) |  |
| C | 2,076 (17.6) | 1,461 (19.6) | 615(14.2) |  |
| **Pathological type** |  |  |  |  |
| HCC | 13,003 (93.0) | 7,347 (95.7) | 5,656(89.7) | <0.001 |
| ICC | 607 (4.3) | 193 (2.5) | 414(6.6) |  |
| CHC | 222 (1.6) | 101 (1.3) | 121(1.9) |  |
| Others | 146 (1.0) | 34 (0.4) | 112(1.8) |  |
| **Tumor nodule** |  |  |  |  |
| Single | 8,610 (80.1) | 5,092 (78.5) | 3,518(82.6) | <0.001 |
| Multiple | 2,134 (19.9) | 1,391 (21.5) | 743(17.4) |  |
| **Tumor thrombus** |  |  |  |  |
| No | 7,263 (65.2) | 4,457 (65.6) | 2,806(64.5) | 0.218 |
| Yes | 3,884 (34.8) | 2,337 (34.4) | 1,547(35.5) |  |
| **Tumor diameter (cm)** |  |  |  |  |
| <3 | 1,497 (12.7) | 957 (12.8) | 540(12.6) | 0.702 |
| ≥3 | 10,281 (87.3) | 6,520 (87.2) | 3,761(87.4) |  |
| **Tumor capsule** |  |  |  |  |
| Yes | 7,405 (69.1) | 4,307 (67.1) | 3,098(72.0) | <0.001 |
| No | 3,314 (30.9) | 2,107 (32.9) | 1,207(28.0) |  |

Data are shown in n (%).
